# Who expands the human creative frontier with generative AI: Hive minds or masterminds?

**DOI:** 10.1126/sciadv.adu5800

**Published:** 2025-09-03

**Authors:** Eric B. Zhou, Dokyun Lee, Bin Gu

**Affiliations:** ^1^ Questrom School of Business, Boston University, Boston, MA 02215, USA.; ^2^ Computing & Data Sciences, Boston University, Boston, MA 02215, USA.

## Abstract

Artists are rapidly integrating generative text-to-image models into their workflows, yet how this affects creative discovery remains unclear. Leveraging large-scale data from an online art platform, we compare artificial intelligence (AI)–assisted creators to matched nonadopters to assess novel idea contributions. Initially, a concentrated subset of AI-assisted creators contributes more novel artifacts in absolute terms through increased output—the productivity effect—although the average rate of contributing novel artifacts decreases because of a dilution effect. This reflects a shift toward high-volume, incremental exploration, ultimately yielding a greater aggregate of novel artifacts by AI-assisted creators. We observe no evidence of a human-AI effect above and beyond the productivity effect. The release of open-source Stable Diffusion accelerates novel contributions across a more diverse group, suggesting that text-to-image tools facilitate exploration at scale, initially enabling persistent breakthroughs by select “masterminds,” driven by increased volume, and subsequently enabling widespread novel contributions from a “hive mind.”

## Teaser

Text-to-image AI boosts idea discovery through high-volume exploration, initially concentrated then broadening to a wider group.

## INTRODUCTION

Artificial intelligence (AI) has emerged as a tool for enhancing human productivity in coding tasks (*1*), knowledge work (*2*), and writing assignments (*3*), with projections indicating substantial economic value for years to come (*4*). In particular, text-to-image generative algorithms based on the latent diffusion model (*5*), such as Stable Diffusion, Midjourney, DALL-E, Imagen, and Flux, have gained traction in creative fields for creating high-fidelity digital artworks based on artists’ ideation. New tools like ControlNet (*6*), image-to-image (*7*), and depth-to-image (*8*) offer users increasingly granular control over the text-to-image pipeline, transforming what was once a labor-intensive process involving human ingenuity and manual skill into a mechanical process guided by human ideas and sense-making (*9*, *10*).

Since the inception of text-to-image generative models, society has largely been concerned with the consequences of such technology on creativity, which is considered a very “human” form of intelligence and expression. Opposition to AI cites that the tool is devoid of the intentionality of human inputs to create anything beyond generic recombinations of existing concepts. Current research on the role of generative AI for ideation is characterized by mixed views on the matter. Early findings suggest that generative AI can be used for rapid idea generation that is both more economical and often higher quality than those produced by humans (*11*) while also being more feasible as evaluated by experts (*12*). On the other hand, there is substantial evidence (*13*) that the ideas produced by generative AI are notably more homogeneous (*14*, *15*). When comparing human and AI on creative benchmarks, findings show that generative AI is capable of producing increasingly creative responses (*16*), yet the very best human ideas can at least match and often outperform AI-generated ideas (*17*, *18*). Other evidence suggests that AI-assisted creators may be leveraging the tool to engage in heightened idea exploration to help them land on novel concepts (*10*). Together, human-AI joint solutions appear more feasible and valuable than AI alone (*19*).

The computational creativity field distinguishes between two types of novel discovery: psychological (P creativity) and historical (H creativity). P creativity refers to ideas new to the individual, while H creativity represents ideas unprecedented in history (*20*, *21*). Thus, a critical interest is whether the expanding universe of creative artifacts is the result of collective effort by many AI-assisted creators—the hive mind—gradually moving toward new frontiers through occasional H-creative artifacts? Or is it driven by a few exceptional AI-assisted creators—masterminds—making notable leaps by consistently generating H-creative artifacts? To explore this, we first consider whether artists who leverage AI tools are, over time, accelerating the expansion of the idea frontier beyond that of their organic counterparts. If such an acceleration is observed, it naturally leads us to ask: Are AI-assisted creators producing more H-creative artifacts in absolute quantity over time compared to organic counterparts? Further investigation then focuses on whether these contributions are a result of the collective efforts of the hive minds or the concentrated bursts of creativity from the masterminds. Recognizing that even innovative contributions can arise from chance, we then consider whether H-creative artists consistently produce novel artifacts or whether their contributions are by random chance. Last, we seek to understand how the increased productivity afforded by generative AI tools fundamentally shapes these dynamics, prompting the question: How does the increased productivity enabled by generative AI tools affect creators’ H-creativity rates?

Our study uses data from a prominent artist platform, including 31,076 unique creators producing digital art over 27 months from August 2021 to December 2023. This period coincides with the release of mainstream state-of-the-art text-to-image models. We identify individual artists who adopt AI tools, allowing us to compare the evolution of the aggregate idea frontier of AI-assisted artists (treated) relative to that of nonadopters (control). Drawing on the formulation of conceptual spaces (*20*, *22*), we operationalize the idea frontier as the convex hull of focal subject matter in artworks, explicitly separating ideation by humans that occur at the prompting and iteration stages of the creative workflow from execution by AI, allowing us to analyze how the tool can affect human ideation. Because the focus of our study is on humans’ role in ideation as opposed to AI’s impact on visual execution, we extract the core subjects, concepts, and/or ideas conveyed in the artworks in the form of text to construct the idea frontier as opposed to comparing images directly in an image embedding space. This allows us to mitigate AI model–related effects on visual elements, which are subject to unobserved differences across host platforms, checkpoints, parameters, etc. We use an event study difference-in-differences design with a bootstrapped matching approach to account for potential selection issues. The Supplementary text contains implementation details.

Our results reveal that before the release of Midjourney V1 (the first mainstream text-to-image model), AI-assisted creators and nonadopters had comparable idea frontiers in terms of size, number of frontier-defining artworks (operationalized as “vertices” of the convex hull and reflecting H-creative artifacts), and number of unique users owning these vertices. The release of Midjourney in February 2022 shows no immediate effect, but following Stable Diffusion 1.5’s release in October 2022, the AI-assisted creators’ idea frontier undergoes accelerated expansion (i.e., the raw number of divergent artifacts increases) relative to that of the nonadopters. Initially, a few AI-assisted creators drive this expansion, but by late 2023, the number of AI-assisted creators owning vertices far exceeds that of the nonadopters. Further analysis indicates that the top AI-assisted vertex contributors tend to more persistently produce novel H-creative artifacts above and beyond their nonadopter counterparts. Over time, text-to-image assistance enables the less prolific hive minds to contribute novel ideas, gradually taking market share from the masterminds. Additional analysis of the effect of the AI tools on individual artists reveals that the H creativity gains are largely due to the productivity gain due to the use of text-to-image tools. In addition, our analysis suggests that the use of the AI tools allows artists to explore more diverse ideas at the expense of their probability of finding frontier ideas. In summary, text-to-image generative AI first gives rise to persistent masterminds driven primarily by productivity gains enabled by AI tools, followed by the emergence of a diverse hive mind that accelerates the exploration of new ideas.

## RESULTS

### Idea frontier expansion following text-to-image releases

**Fig. 1.**
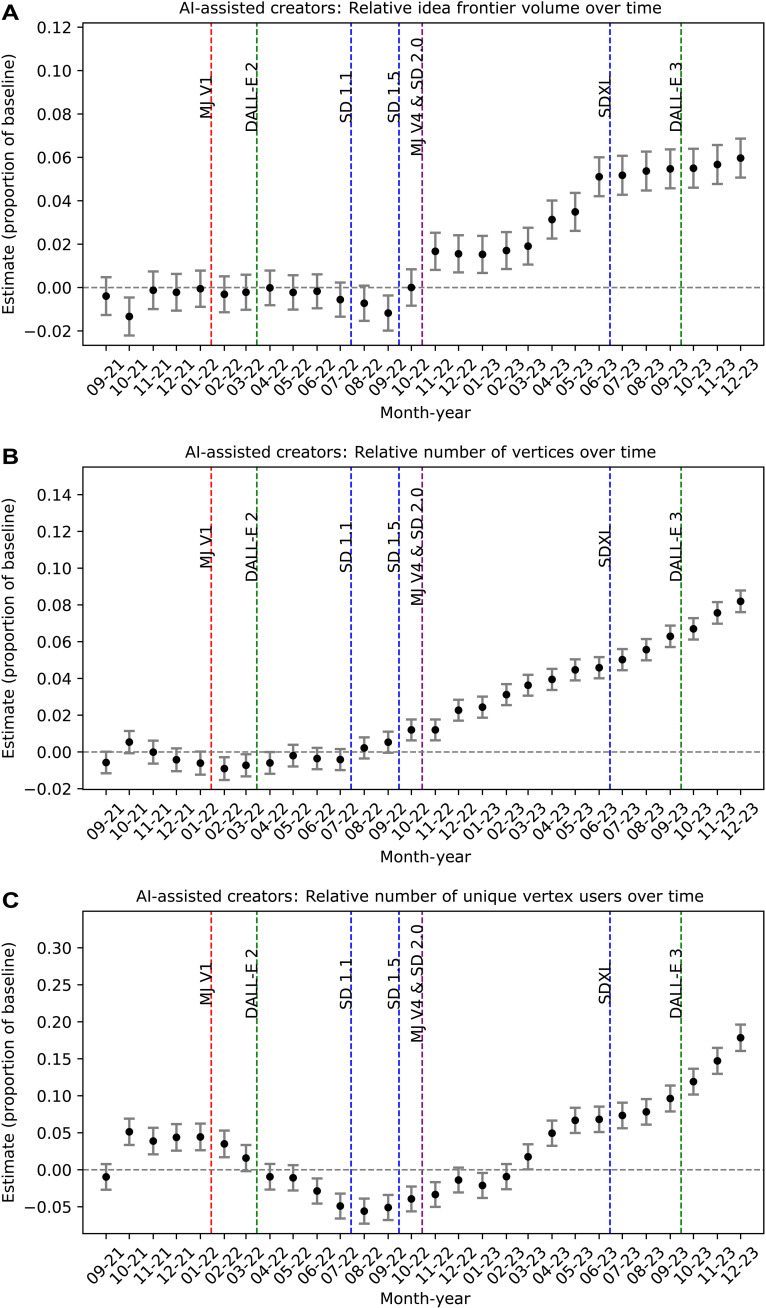
Event study difference-in-differences estimates for the relative gains in AI-assisted creators’ idea frontier metrics. The point estimates measure the AI-assisted creators’ idea frontier gains in (**A**) volume, (**B**) number of vertices, and (**C**) number of unique users who own a vertex on the idea frontier relative to the nonadopters’ idea frontier as a proportion over the baseline in the starting period (intercept plus the treatment fixed effect). A value of 0.1 denotes that the gain in the AI-assisted creators’ outcome over the nonadopters’ resulted in a 10% increase over the baseline. The error bars represent 95% confidence intervals.

Results shown in Fig. 1A present several noteworthy findings. First, the treated AI-assisted creators’ and control nonadopters’ idea spaces show no significant differences before Midjourney V1’s release in February 2022, indicating the effectiveness of our matching procedure in accounting for unobservable cohort differences. Second, the release of Midjourney V1 did not immediately lead to expansion in the treatment group’s idea space, suggesting that early text-to-image models did not necessarily enable AI-assisted creators to surpass nonadopters in idea discovery. This may be attributed to the closed-source nature of Midjourney and DALL-E 2, which only allowed for simple text-to-image creation. Their outputs are likely an artifact of the training data’s “idea universe” rather than enabling creators to exploit niche ideas.

The release of Stable Diffusion 1.5 in late 2022 marked a gradual acceleration in the idea frontier expansion among AI-assisted creators. This open-source model, unlike the closed-source Midjourney and DALL-E 2, potentially contributes to this acceleration by allowing individuals to fine-tune models and explore niche content. Vibrant communities also emerged within which customized models and intricate workflows are freely shared to promote targeted exploration and exploitation. In addition, advances in computer vision and diffusion models, including low-rank adaptations for niche content generation (*23*), ControlNet for interactive image manipulation (*6*), image-to-image techniques for refinement (*7*), and depth-to-image methods for composition synthesis (*8*), may contribute to this acceleration. Such instruments provide AI-assisted creators with unprecedented control over the creation process, enabling them to exploit niche concepts in ways not possible with closed-source models.

Next, we examine the absolute number of vertices defining the idea frontier in each period, as shown in Fig. 1B. Vertices represent instances of exploratory creativity at the edge of the frontier and thus are considered H-creative contributions. The estimates indicate that, compared to the nonadopters, the AI-assisted idea frontier is composed of many more artworks post–Stable Diffusion. We also observe significant volume gains as seen in Fig. 1A. The combination of the two outcomes suggests that the idea frontier is expanding gradually through small, incremental contributions rather than substantial leaps via radically divergent ideas.

### Attributing expansion to the hive mind or masterminds

We next observe the number of unique creators who contributed a vertex artwork, representing the concentration of H-creative artists within each cohort. Figure 1C reveals that the number of unique H-creative artists is relatively stable but larger among AI-assisted creators compared to the nonadopters before any model is released. This likely results from the matching process affecting user composition rather than directly matching on the initial idea space. To address this limitation, we use the Synthetic Control method estimated over the set of unique nonadopter idea spaces as a robustness check (see the “Robustness Checks” section).

Between 2022 and 2023, the composition of H-creative artists becomes more concentrated among AI-assisted creators, indicating that a few exceptional artists drive the early expansion of the idea frontier. This trend reverses in 2023, suggesting that AI-assisted creators are collectively engaging in more exploration of the idea frontier, signaling the emergence of a “hive mind.” By the end of 2023, the number of H-creative AI-assisted creators exceeds the nonadopter baseline, implying that more creators are now able to contribute novel ideas. This pattern suggests that early AI-assisted creators may experience a period of adjustment as they learn to leverage new tools and best practices, potentially leading to complementarities between human and AI capabilities in subsequent interactions, enabling a greater number of AI-assisted creators to produce a wider range of exploratory artifacts (*24*). This early concentration may have been driven by early adopters leveraging AI tools for high-volume production.

### AI-assisted creativity as a stochastic versus persistent process

To determine whether novel idea contribution is a stochastic or persistent process, we analyze the H-creative artists who contribute vertex artworks to the idea frontier and assess their ability to replicate this over time. For the 1000 AI-assisted creator and nonadopter cohort permutations, we identify the unique users with a vertex in any month, calculate their total vertex contributions per period, rank them by total contributions, and aggregate the count of vertex contributions by rank deciles. This allows us to create decile-month panels for both AI-assisted creators and nonadopters, yielding the average number of vertex contributions per decile over time.

**Fig. 2.**
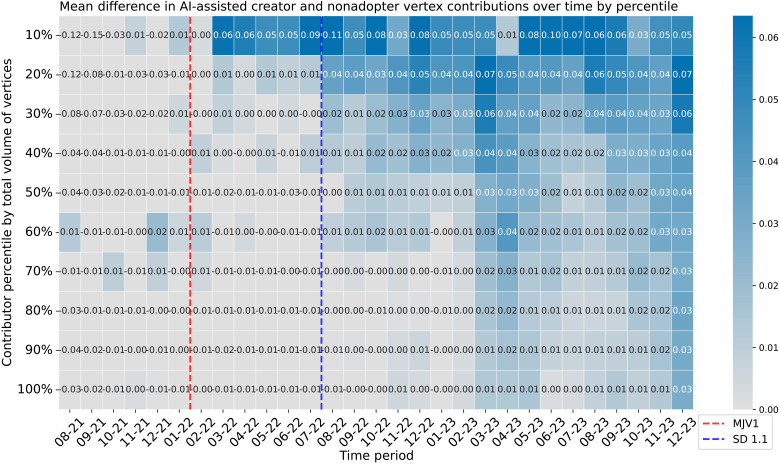
Heatmap for the average difference in number of vertex contributions between AI-assisted creators and nonadopters grouped by percentiles of total vertices contributed. The number in the cells denotes the average difference in the number of vertex contributions between AI-assisted creators and nonadopters in that period, and the intensity of the hue indicates the degree of difference.

We show the difference between the AI-assisted creators and nonadopters’ average vertex contributions in Fig. 2. Observe that before the release of Midjourney in February 2022, differences between the AI-assisted creators and nonadopters across rank deciles are negligible or negative. This suggests that AI-assisted creators were on par or not performing relative to their nonadopter counterparts during the pre-Midjourney period. The negative difference arises because deciles are based on total contributions over the entire data period, rather than focusing solely on users who were more productive before text-to-image tools. By grouping deciles based on total vertex contributions, we better capture users who become increasingly productive after Midjourney’s release, which is more indicative of the role generative AI plays in fostering persistent H-creative activities.

Following the release of Midjourney, most vertex contributions are concentrated among the top 10 to 20% of AI-assisted creators compared to nonadopters, a trend that persists throughout the observation window. This suggests that top creators, or the “masterminds” henceforth, become more consistent H-creativity contributors with AI assistance. Among the lower 80% of contributors, which we define as the hive mind, there is a noticeable lag in their ability to contribute vertex ideas that may be partially explained by variation in adoption timing. While early differences between AI-assisted creator and nonadopter groups are minimal, the adoption of text-to-image tools seems to provide creators with an increased likelihood of making novel contributions over time. This effect is inconsistent for lower-ranked users, indicating that beyond the top 20% of users, contributions are generally stochastic or one-off discoveries, particularly as the idea space becomes saturated. The lowest-ranked contributors exhibit this stochastic pattern of contribution even in later periods.

Next, we analyze the total market share of vertices owned by users in each rank percentile to understand how contribution patterns may have led to the emergence of the hive mind. By averaging the volume of contributed ideas relative to the total number of ideas in each period, we calculate the share of vertex ideas belonging to each rank percentile across four distinct periods: before text-to-image AI, post-Midjourney but pre–Stable Diffusion 1.5, post–Stable Diffusion 1.5, and the last six months of 2023, to capture the long-term state of the idea frontier.

**Fig. 3.**
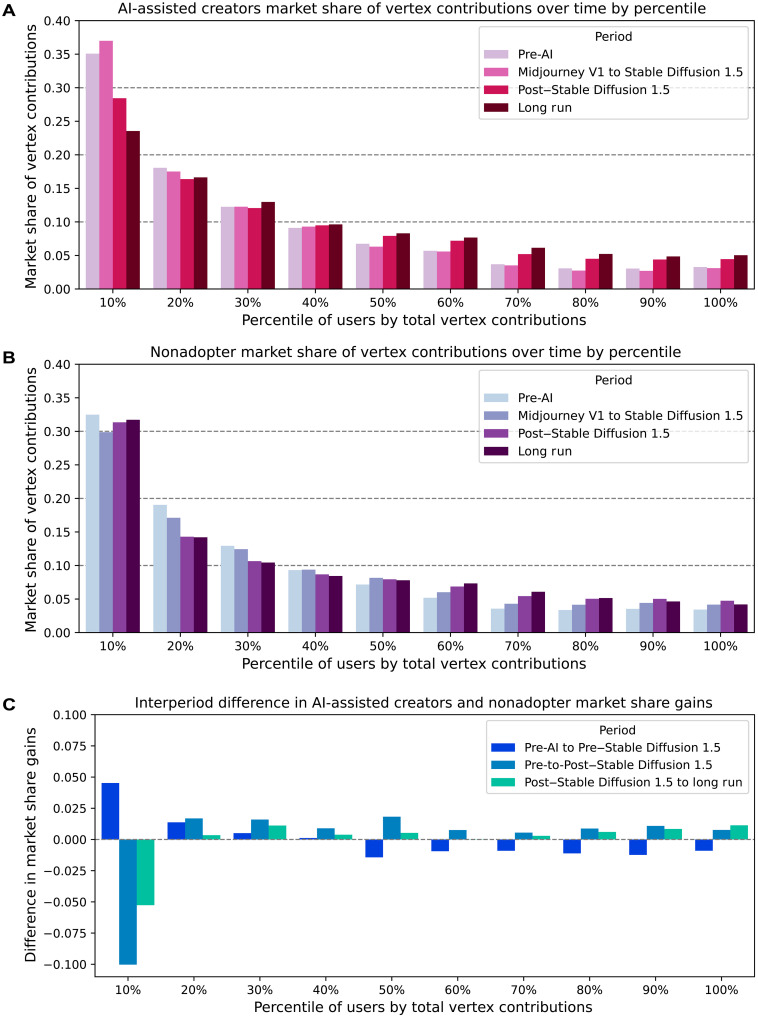
Market shares grouped by user percentiles of total vertices contributed. (**A** and **B**) depict per-period market share of vertices for (A) AI-assisted creators and (B) nonadopters within pre-AI, post-Midjourney, post–Stable Diffusion 1.5, and long-run time periods. (**C**) compares interperiod differences in AI-assisted creators’ and nonadopters’ market share gains of vertices owned across pre-AI, pre–Stable Diffusion 1.5, post–Stable Diffusion 1.5, and long-run time periods grouped by percentile of total vertices owned. Each bar indicates the aggregate difference between AI-assisted creators and nonadopters in their market share gains between periods. A value of 0.05 for the pre-AI to pre–Stable Diffusion 1.5 period indicates that the AI-assisted creators, on average, gained 5% more market share than their nonadopter counterparts when comparing their pre-AI to pre–Stable Diffusion 1.5 shares.

Examining market shares among the AI-assisted creators in Fig. 3A, we see that the top 10% of creators hold nearly 35% of all vertex artworks in the pre-AI period, with this concentration increasing with the release of Midjourney. This indicates that a select set of prominent creators can dominate the idea space even without AI assistance, but AI assistance further cements their dominance. Following Stable Diffusion 1.5, the share of vertices begins to disperse among lower-ranked users. By the end of 2023, the top 10% hold approximately 23% of the total vertex share, a decrease of 12% from their initial market share, as lower-ranked users successfully contribute a greater share of novel ideas. This suggests the emergence of a stochastic hive mind that can potentially make meaningful contributions to the idea frontier when aided by text-to-image tools. In contrast, the nonadopters, as shown in Fig. 3B, consistently see the top 10% of users maintain more than 30% of all vertex contributions throughout the observation window, with minimal fluctuations in their dominance.

We plot the difference in interperiod gains between AI-assisted creators and nonadopters in Fig. 3C. The results indicate that the top 10% of AI-assisted creators gain a sizable portion of market share following Midjourney relative to the nonadopters, while users in the 20th and 30th percentiles experience only minor gains compared to their nonadopter counterparts. However, the top 10% creators lose a large portion of their market share with Stable Diffusion 1.5 and by the end of 2023 relative to the nonadopters. For creators in the 50th percentile and below, the release of Midjourney initially results in a loss of market share, but gradually, these users capture share from the top creators. This trend suggests a collective improvement among creators over time, culminating in a long-run hive mind.

Overall, these results reveal that creativity, irrespective of AI usage, is marked by the persistence of select masterminds who can replicate their successes over time. With the introduction of generative AI tools, these dominant creators can consistently produce increasingly novel ideas compared to nonadopters. Furthermore, AI assistance facilitates the emergence of a long-term stochastic hive mind that, while unable to consistently generate novel ideas, can still make meaningful contributions through what appear to be serendipitous discoveries.

### Productivity effects, human-AI effects, and dilution effects

While the absolute number of H-creative occurrences is increasing for the AI-assisted creators relative to their nonadopter counterparts as seen in Fig. 1B, what about the rate of H creativity? This raises the question: To what extent is the expansion of the idea frontier driven by the increased productivity of AI adopters, allowing for more attempts at finding new ideas, versus a genuine complementarity between human and AI, enabling their joint efforts to generate H-creative artifacts?

In particular, when considering the overall effect of generative AI tools and idea discovery, there are a few dynamics possibly at play. First, AI can simply be a tool to automate visual execution, allowing users to realize all potential combinations of known concepts in their repository meanwhile experiencing substantial productivity gains. Thus, one benefit is the increased productivity effect: By simply allowing creators to realize all of their own concept combinations, they can potentially contribute something novel driven by sheer statistical chance. Second, AI can serendipitously reveal previously overlooked concepts to humans by allowing them to explore their personal idea space while the human can exercise their own control within the creation process with tools and workflows, allowing human and AI to jointly reach a novel solution in a synergistic manner. This interaction, which we call the human-AI effect, allows the human to tap into cultural norms encoded in the model during training to navigate the known set of concepts and expand their personal repository of concepts to arrive at an output that surpasses that of human or AI alone. Conversely, previous research has demonstrated that individual-level productivity gains among creators leveraging text-to-image models are accompanied by a decrease in average idea novelty, suggesting a tendency toward producing more generic artifacts (*10*). This outcome reflects a dilution effect—a creative regression to the mean with respect to idea novelty—driven by heightened productivity enabled by AI tools. Consequently, the rate at which frontier-expanding artifacts emerge may decline after adoption.

The impact of the productivity effect on creators’ ability to find new ideas—or H creativity—could lead to three potential outcomes. First, if the vertex discovery rate of AI-assisted creators decreases as productivity increases, this might suggest that these individuals’ behaviors are shifting toward less intentional or deliberate creative processes as they simply mass produce content, reflecting the dilution effect. Second, if the rate of new idea discovery increases with productivity, it could indicate a complementarity from the interaction between creators and AI—the human-AI effect—where increased productivity combines with a synergy between artist and AI to more effectively generate innovative ideas. Last, a stable rate of idea discovery would imply that AI-assisted creators perform on par with those using traditional tools—indicating neither a detectable human-AI complementarity effect nor a dilution effect—while preserving a similarly deliberate approach to novelty generation when working with AI.

To disentangle the productivity and human-AI effects, we reestimate the convex hulls of the AI-assisted creators assuming that they produce the same raw number of artifacts as the nonadopters in the post-Midjourney period. This adjustment accounts for the possibility that AI-assisted creators are contributing vertex artifacts solely due to increased productivity. The difference in the number of vertices that characterize the convex hull between the productivity-equalized and nonproductivity-equalized results quantify the vertex discovery rate accounted for by the productivity effect. We also examine underlying heterogeneity in vertex discovery rates between the identified masterminds and hive minds as well as between users who are minimally and highly exploratory before the adoption of AI tools. Materials and Methods contains implementation details.

**Fig. 4.**
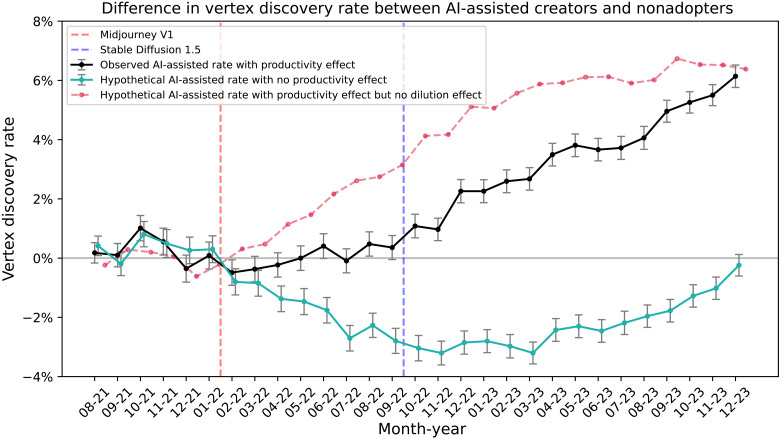
Difference in vertex discovery rate between AI-assisted creators and nonadopters. Depicts the gains in AI-assisted creators’ vertex discovery rate with the productivity effect (black) and without the productivity effect (teal) relative to the nonadopters. The red line denotes the hypothetical vertex discovery rate if AI-assisted adopters maintained their H-creativity rate in the absence of the dilution effect while also experiencing the productivity effect.

Figure 4 reveals that by adjusting for productivity effects, the rate at which AI-assisted creators contribute vertex artifacts is lower than that of their nonadopter counterparts. Once AI-driven productivity gains are removed, the observed (black) vertex discovery rate declines to the hypothetical (teal) rate, indicating that AI-assisted production leads users to produce new ideas less selectively and intentionally, thereby lowering the baseline H-creativity rate when leveraging text-to-image tools. The reduction in found vertices after the release of Midjourney V1 corresponds to an average 2.1% fewer frontier-defining artifacts over the entire set of vertices over time. Without adjusting for the productivity effect, note that the trend mirrors the main result for the number of vertices over time in Fig. 1B. The gains from the productivity effect correspond to 2.2% gains in the number of found vertices on average post-Midjourney V1. Consequently, the net increase in vertex discoveries attributable to the productivity effect is approximately 4.3% greater vertex artifacts contributed after Midjourney V1, with a maximum gain of 6.6% in new vertices found by the end of 2023. There appears to be a trade-off between increased productivity and the ability of AI-assisted creators to explore new ideas effectively. The red line depicts the hypothetical vertex discovery rate the AI-assisted creators would have achieved had they sustained their H-creativity rate while still benefiting from AI’s productivity gains. In summary, AI-assisted creators contribute more H-creative artifacts in absolute terms through increased output (the productivity effect), although the average rate of H-creative artifacts falls because of the dilution effect. We observe no evidence of a human-AI effect above and beyond the productivity effect.

**Fig. 5.**
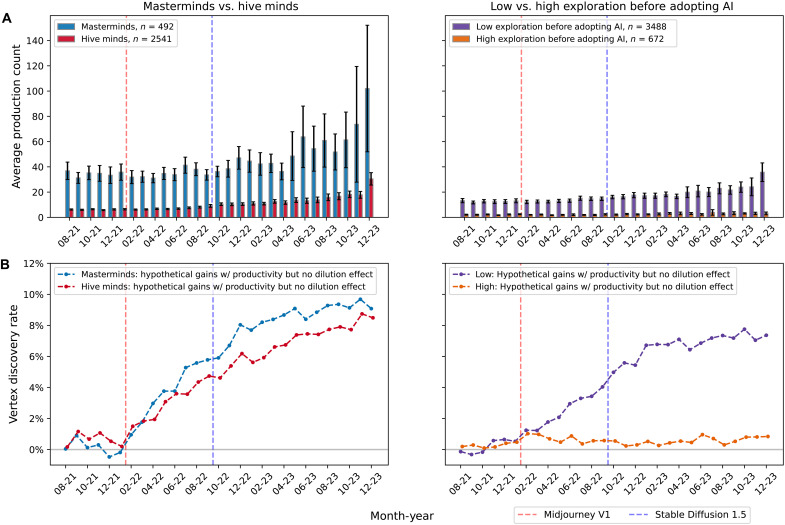
Average productivity and impact of the productivity effect on vertex discovery rates across cohorts. (**A**) Average raw productivity over time for (left) masterminds and hive minds and (right) low- and high-exploration users before adopting AI. (**B**) Hypothetical gains in vertex discovery rate with the productivity effect and without the dilution effect for (left) masterminds and hive minds and (right) low- and high-exploration users before adopting AI.

We next focus our analysis on how individual-level differences may explain differential gains from the productivity effect. Recall that the masterminds are the top 20% of total vertex contributors who consistently contribute frontier-expanding ideas, whereas the hive minds are made up of the remaining bottom 80% of users who make more stochastic contributions. Figure 5A shows that the AI-assisted masterminds are inherently much more productive compared to the hive minds, producing 4.4 times more artifacts on average. For comparison, we replicate the red hypothetical-line procedure from Fig. 4 to estimate counterfactual vertex discovery rates for masterminds and hive minds under AI’s productivity effect alone (i.e., excluding the dilution effect), as shown in Fig. 5B. The figure depicts that masterminds experience a greater gain in their vertex discovery rate compared to the hive minds, largely due to the magnitude difference in productivity. These results suggest that individuals identified as masterminds may simply be prolific creators who potentially benefit from the human-AI effect through the sheer scale at which they can explore. These patterns echo established regularities in art history: Picasso’s dominance by sheer volume (*25*), a power-law concentration among top artists (*26*), and renowned painters attaining status through disproportionately high output (*27*).

Next, we examine how creators with different exploration tendencies before AI tools benefit from the productivity effect after adopting AI tools. As shown in Fig. 5A, AI-assisted creators who were initially less exploratory appear to leverage text-to-image AI to drastically increase production compared to those who were highly exploratory before adopting—a group that seems to experience less enhancement in productivity after adopting AI. Consequently, AI-assisted creators with low initial exploration benefit substantially from the productivity effect, as their rate of new idea discovery increases dramatically with productivity gains, as illustrated in Fig. 5B. Conversely, AI-assisted creators with high initial exploration do not appear to benefit from the productivity effect, as their production volume remain largely unchanged. This might suggest that highly exploratory creators remain as deliberate in their creative process as when they did not use AI tools, allowing them to maintain their H-creativity rate.

In summary, the productivity effect appears to be the primary driver behind AI-assisted creators’ ability to find novel ideas. There is no evidence of the human-AI effect; instead, the dilution effect appears to hinder creators’ vertex discovery probability when productivity gains are not realized. This suggests that while adopters readily realize the first-order benefits of generative AI through productivity effects, substantial opportunities remain to enhance ideation rates without incurring dilution effects, thereby unlocking the potentially transformative human-AI synergy that serves as a critical frontier for future research.

### Robustness checks

A core concern regarding our main identification strategy is that while we attempt to construct similar AI-assisted creator and nonadopter cohorts by matching on observable user characteristics like their average number of publications before Midjourney and platform prominence as proxies for creator quality, we cannot directly match on the initial convex hulls. This means that even if the composition of users within the matched AI-assisted creator and nonadopter cohorts are comparable in terms of observable traits, their actual idea spaces can be quite different in terms of size, number of vertices, and number of unique users owning those vertices. Thus, there are unobservable variables that we are not accounting for, such as idea diversity within the population.

To explicitly address this concern, we leverage the 1000 unique permutations of the AI-assisted creators to first generate a single aggregate treatment idea space and then use the nonadopter cohorts to apply the Synthetic Control method (*28*) where we create a counterfactual control idea space as the weighted combination of the set of precomputed nonadopter idea spaces. Doing so allows us to recover a counterfactual idea frontier that resembles the single aggregate AI-assisted creators’ idea frontier in terms of size, number of vertices, and number of unique vertex users in the pre-Midjourney period. Specifically, we use the augmented Synthetic Control approach (*29*), which is an extension of the foundational Synthetic Control method that leverages a ridge regression outcome model that simultaneously controls for pretreatment fit while also minimizing extrapolation from the convex hull defined by the nonadopters. This approach will recover weights that are at least as good in pretreatment fit as the standard Synthetic Control method while offering some computational benefits given the large number of nonadopters to optimize over.

**Fig. 6.**
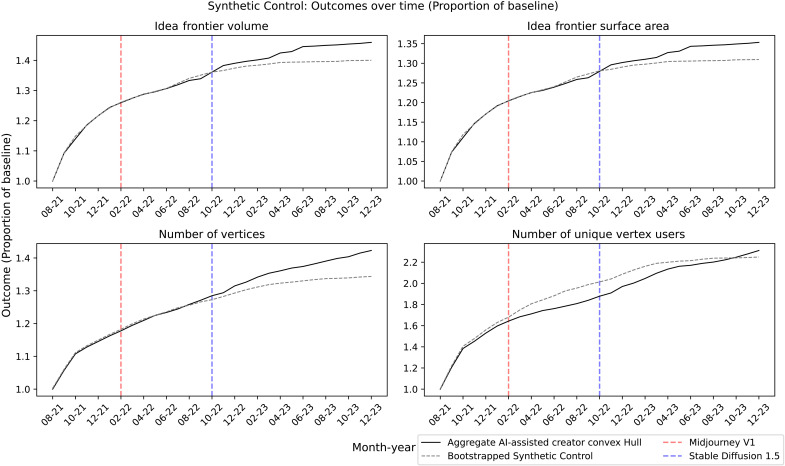
Synthetic Control method estimates for main outcome variables. The AI-assisted creators’ outcomes are aggregated as the average over all unique permutations of the convex hull. For 100 bootstrapped iterations, 200 unique nonadopter convex hulls are sampled to construct the Synthetic Control unit optimized over the period before Midjourney V1.

For each of the main outcome variables, we use a bootstrapping procedure where we first take the aggregate AI-assisted creators’ idea space as the average across all 1000 permutations of the treatment group. Then, for 100 iterations, we take a random sample of 200 nonadopter idea spaces and compute the weight matrix for the counterfactual unit using the size, number of vertices, number of unique vertex users, and number of users composing that idea space (since the matching procedure may drop a few users who are not successfully matched). The pretreatment fit is optimized over the period until the release of Midjourney in February 2022. Using the recovered weight matrix, we then compute the outcome for the Synthetic Control unit as the average over each of the 100 iterations for each outcome. The results are plotted in Fig. 6.

The figures reveal that when performing pseudo “matching” on the pretreatment idea space with the Synthetic Control method, the results are consistent with the main estimation procedure. Specifically, the pretreatment outcomes are virtually identical between the AI-assisted creators and Synthetic Control. For the volume, surface area, and number of vertices, we observe the divergence in outcomes at the time of Stable Diffusion 1.5, once again consistent with the main results. With regard to the number of unique vertex users, the trend resembles that of the main result where there is an initial concentration in the number of vertex owners post-Midjourney followed by a diversification after Stable Diffusion 1.5. The pretreatment fit also appears improved over the main result, with the divergence between the AI-assisted creators’ and Synthetic Control idea spaces occurring within proximity to Midjourney’s release.

We also consider the possibility that some users who are actually AI-assisted creators choose not to disclose their treatment status, thus contaminating the nonadopter control group. To assess the robustness of our treatment effect estimates in the presence of misidentification of adopters, leading to control group contamination, we run a sensitivity analysis that allows for varying degrees of unidentified treatment spillover into the control group. We confirm that our results are robust up to 30% contamination of the control group with unidentified adopters. Further, this level of contamination merely attenuates our estimates by approximately 50%, suggesting that our current results are a conservative estimate of the true effect. See the Supplementary Materials for details.

## DISCUSSION

As generative AI has emerged as this generation’s general purpose technology with the potential to broadly disrupt the economy (*30*), many empirical works specifically investigate whether and when humans outperform AI in creative tasks (*11*–*19*). Results are mixed, with some studies suggesting that humans surpass large language models at generating novel, high-quality ideas, whereas others find that generative AI is at least on-par with human performance under certain conditions. Current discourse proposes that AI can catalyze divergent thinking, helping us to find solutions that are novel, valuable, and potentially unforeseen by humans (*31*).

Specifically focusing on how text-to-image AI may be augmenting humans’ ideation to land on novel and unforeseen solutions, we find that AI-assisted creators can accelerate novel idea contributions with the release of the open-source Stable Diffusion, outperforming nonadopters. In addition, the initial release of closed-source Midjourney led to a concentrated group of masterminds that persistently contribute most new ideas. Over time, the pool of unique AI-assisted contributors diversifies as a hive mind emerges, making stochastic contributions to the idea frontier with the introduction of open-source models and tools—a result analogous to the discovery of new strategies by human players in the game of Go following the release of AI system AlphaGo (*32*). The evidence suggests that generative AI enhances creators’ likelihood of contributing novel ideas in the long run, even as the idea space becomes saturated. Thus, by lowering the barrier to entry, AI assistance has the potential to broaden the population of artists capable of contributing novel ideas that can introduce greater diversity to the artifacts being produced (*33*).

A useful theory to consider for the underlying mechanisms is blind variation and selective retention, which frames creativity as sampling new ideas (variation) from an idea distribution and selecting the most promising ones (selective retention) (*34*). Variation is characterized by its blindness condition, suggesting that it is not constrained by specific goals, while the overall creative process can involve evaluating outputs against selection criteria within a genetic algorithm framework, where prior steps inform future iterations (*35*). Thus, the creative process is structured as ideation, execution, curation, and iteration in an evolutionary framework.

One possibility is that the text-to-image paradigm automates the visual execution of an idea without necessarily affecting the humans’ ideation process. The creator remains the originator of the idea and is entirely responsible for expressing concepts through prompting. Design choices, such as model checkpoints, correspond to realizing specific visual features. With closed-source models, AI explicitly separates ideation from implementation, allowing creators to invest additional effort into realizing existing ideas or experimenting with concepts without concern for resource constraints. This reduction in technical barriers along with the productivity benefits—or productivity effect—may encourage further idea exploration at scale, while artistic style primarily reflects models’ training data. Ideas generated through this combinatorial process can be distinct creative contributions through the sheer scale of exploration, ultimately facilitating the expansion of the idea frontier. However, creators may become less intentional in their creative process when they begin using AI tools, leading to a dilution effect where the increased production leads to a lower rate of contributing novel artifacts.

Another possibility is that text-to-image workflows facilitate ideation and iteration, allowing model randomness in generation to yield unexpected outputs or even serendipitous discoveries. This enables creators to sample from their idea distributions and map specific words or concepts to potential visual realizations with minimal effort—essentially mapping their individual idea space and exploring the correspondence between their concepts and possible executions. Given that prompting and idea mapping are inherently verbal exercises, there is likely a complementarity between humans’ verbal fluency and text-to-image model capabilities—or the human-AI effect. Recent research has shown that individual cognitive abilities and more expressive vocabularies, rather than AI alone, can lead to more creative AI-assisted artifacts (*36*). This collaborative process can inspire creators to exploit interesting concepts derived from this mapping. Subsequently, they can use instruments available for open-source models to intricately refine these concepts, potentially resulting in novel creations. Therefore, this creative process involves human-AI interaction to explore idea variations, followed by the machine executing and mutating the initial idea with human assistance, and concluding with human filtering among a diverse set of possibilities (*37*).

Mechanically, text-to-image models map a text-based “idea space” to a visual “solution space.” Solutions in this visual space can be seen as convex combinations of existing visual-concept pairs from the training data (interpolative), with potential for novel explorations beyond the convex hull of known knowledge (extrapolative) (*38*, *39*). Closed-source models that limit exploration to simple text-to-image processes are constrained by their fixed knowledge base. In contrast, open-source models that allow intimate human interaction encourage exploration beyond the constrained solution space. Early closed-source models, while enabling rapid production, arguably only generate interpolative artifacts—combinations that remain within the established universe of ideas, as reflected in our results. With open-source models, creators can exercise control over idea exploitation, allowing them to escape a constrained solution space, a shift evident with Stable Diffusion.

**Fig. 7.**
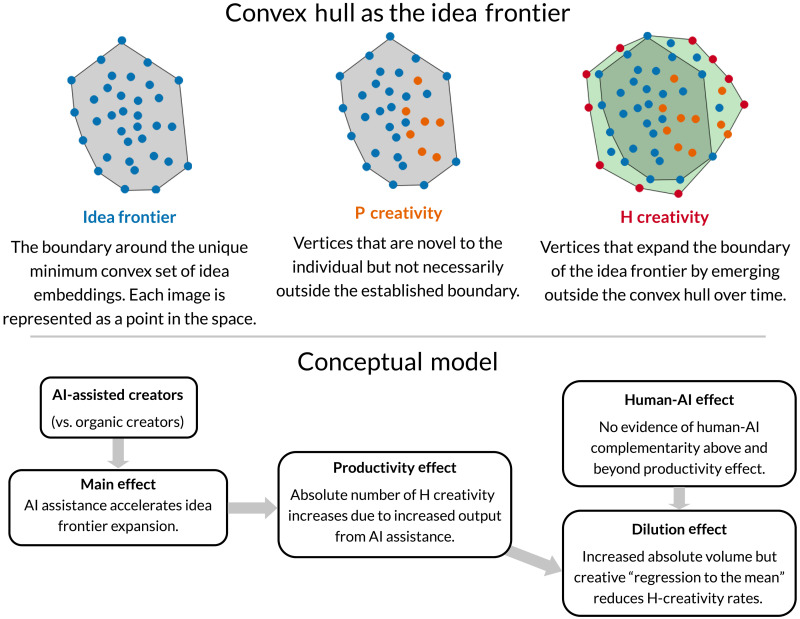
Conceptual diagram. Definitions for key concepts (idea frontier, P creativity, and H creativity) and a conceptual model illustrating the main effect and mechanisms (productivity, human-AI, and dilution effects).

While both effects may be at play, we find no evidence of the human-AI effect on H creativity in our setting. In most cases, creators take known concepts and fill in the idea space with largely combinatorial contributions, as evidenced by the comparable idea frontier sizes between AI-assisted creators and nonadopters before Stable Diffusion. There still remains the possibility that with open-source models, creators can actively participate in a hands-on iterative process to find, exploit, and refine ideas that emerge from the text-to-image workflow to engage in exploratory creativity. As new open-source models, training data, and instruments are released, we can expect the repository of ideas in the training data represented within the models to expand and grant creators greater control over the exploration and exploitation process. AI-assisted creators only appear to improve their H-creativity rate if they experience the productivity benefits of AI, suggesting that the human-AI effect may coincide with the productivity effect but not in isolation. Without the productivity effect, AI-assisted creators seem to experience a dilution effect when leveraging AI tools where, on average, their creativity regresses to the mean, reducing their vertex discovery rate despite the absolute number of vertex artifacts increasing. This insight poses an interesting avenue for future research. Figure 7 provides an overview of the key concepts, main effects, and mechanisms in this study.

We acknowledge several limitations of our study. First, our sample comes from a digital art platform catering to hobbyists as opposed to industry professionals or artists working in physical or offline mediums. Consequently, it will be valuable to assess the generalizability of our findings to other artist communities and creative communities in general. Second, while novelty is a key element of creativity, our study does not address whether all instances of vertex ideas are meaningful or accepted contributions to the broader artistic community. H creativity, as defined in the literature, explicitly pertains to novelty rather than value (*20*). Consistent with this definition, our operationalization of H creativity also emphasizes novelty, and consequently, our convex hull measure inherently does not incorporate assessments of value. Thus, combinational innovations, such as Picasso’s synthesis of Western and African art, may offer substantial value despite not appearing as extreme points. We acknowledge that this represents an important limitation of our measurement approach, as some meaningful forms of creativity may remain undetected within the interior of the idea space. Whether novelty generates value, and if so, under what conditions, remain important open questions for future research. Third, while we consider the direct interaction between human and AI, there is also likely across-user idea transfer and learning from peers and AI that underlies idea discovery. An interesting question for future research is to what extent a community’s social fabric facilitates idea discovery via learning. Lastly, while we use multiple causal inference techniques—including propensity score matching, bootstrapping, and the Synthetic Control method to strengthen the causal interpretation, we acknowledge the possibility of unobserved confounders. Therefore, our findings should be interpreted as suggestive of a causal relationship, rather than definitive proof. Future research should prioritize identifying and addressing potential unobserved factors. To address potential concerns, we perform sensitivity analyses accounting for the impact of unobserved confounders, such as idea diversity and control group contamination. These analyses indicate that our primary conclusions remain robust even under suboptimal conditions; however, we encourage future studies to rigorously investigate potential unobserved factors and establish causal inferences.

In summary, our findings reveal that text-to-image generative AI has the potential to fundamentally transform creative exploration by enabling idea discoveries beyond traditional boundaries. The emergence of open-source tools and community-driven innovations has democratized creative control, empowering artists to potentially transcend the limitations of model training data through sophisticated manipulation of the generation process. This transformation exemplifies “generative synesthesia” (*10*), where human creativity and AI capabilities merge to produce breakthrough outcomes through novel workflows. By shifting artistic focus from technical execution to ideation, curation, and refinement, both established and emerging creators can achieve creative breakthroughs, enabled primarily by the productivity gains enabled by text-to-image tools. The sustained expansion of the idea frontier, driven by both persistent masterminds and an emerging hive mind, demonstrates that generative AI can act not as a constraint but as a catalyst for human creative expression, although high-volume production appears necessary to reap the full benefits of AI tools; otherwise, creators may experience a regression to the mean by using AI. Rather than stagnating creativity, AI tools are expanding the universe of possible ideas, suggesting a future where technology amplifies rather than diminishes human creative potential in aggregate.

## MATERIALS AND METHODS

### Identifying AI adopters

Platform policy often encourages users to disclose AI assistance through artwork tags. For artworks predating January 2021 (the original DALL-E release), we automatically classify them as non-AI. For newer works, we first analyze post titles and tags for keywords like “AI-generated,” “Stable Diffusion,” “Midjourney,” and “DALL-E.” This identifies artworks where users explicitly acknowledge AI use. Further, we also monitor AI art communities within which only known AI-assisted artifacts are published, which is enforced by the platform. We compile these artworks that either contain an AI-related tag and/or are published in an AI art community and label them as AI generated. Users with any of their publications identified as AI assisted is considered a treatment unit, whereas any user who has never published an artifact with AI assistance is then a control unit. We use the earliest identified publication date for each treatment user as the adoption date.

### Idea feature extraction

A noteworthy result from (*10*) is evidence that AI-assisted creators’ idea frontier is expanding marginally faster than that of nonadopters. We first expand on this result by operationalizing and explicitly estimating the idea frontier. Second, the previous result leads to an immediate follow-up question: Is the expanding idea space being driven by few mastermind adopters exploring the frontiers or is generative AI enabling adopters as a whole to jointly expand the idea frontier? We explore this dynamic by focusing on the idea space characterized by the meaning or subject matter in creative artifacts as suggested by the philosophy of symbolism (*40*, *41*).

To identify the focal objects and relationships in an artifact, we use the multimodal model BLIP-2 (*42*), which takes as input an image and outputs a text description while also allowing for controlled text generation to produce more stable descriptions that are consistent in length and structure. This approach avoids errors that may be contributed by cross-individual differences at the user level. We then leverage a BERT-based text embedding model fine-tuned for semantic similarity search (*43*), which has demonstrated state-of-the-art performance on semantic similarity benchmarks while also being highly efficient, to compute high-dimensional vector representations for each extracted idea.

### Operationalizing the idea frontier

Expansion of the idea space can be attributed to three related concepts as proposed by (*20*). First, humans can engage in P creativity, which denotes instances when an individual produces something that they themselves have not produced in the past. Second, instances of P creativity that have also never before been created by all of humanity can be defined as H creative, which is a special type of P creativity. Thus, any scenario in which the idea frontier is expanding is evidence of both P-creative and potentially H-creative production. Third, the idea space can be captured by a conceptual space, which is a structured geometric representation of entities along which different dimensions capture particular attributes of the entities (*20*, *22*).

Together, we can think of the idea space as the minimal boundary encapsulating all existing ideas. Naturally, this is analogous to the convex hull, which given a set of points *X* in a Euclidean space, is formally defined as the unique minimum convex set containing *X*. In our setting, this corresponds to *X* being the set of all artwork idea embeddings and the idea frontier being the minimal convex set that captures *X* and the vertices of the idea frontier being the extreme values along the attributes that define the idea space. Thus, we operationalize creative expansion as the expansion of the convex hull of artworks’ core ideas over time.

To accomplish this, we must first reduce the dimensionality of the extracted 384-dimension idea embeddings such that computing the convex hull is computationally feasible over large samples of artworks. We leverage a state-of-the-art nonlinear dimensionality reduction algorithm Uniform Manifold Approximation and Projection (UMAP) (*44*) to best preserve the information in the high-dimensional space while being highly efficient. UMAP offers noteworthy benefits to modern neural topic modeling approaches because of its ability to preserve the local and global high-dimensional space in the lower-dimensional representation (*45*). For computational complexity reasons related to computing the convex hull, we use the UMAP model to reduce our embeddings to a five-dimensional space as it offers a fair trade-off in representation fidelity while reducing excessive resource demands in computing the convex hulls.

We also instantiate each idea space by including an established set of historical artworks from which we perform our idea extraction procedure such that every idea space begins with the same “universe” of ideas. Intuitively, this is to say that all users have access to the same set of existing ideas, so any individual contributions that do not extend beyond the known set is not considered an H-creative contribution. We leverage the SemArt dataset (*46*), which contains 21,384 image-description pairs that captures the semantic contents of fine-art paintings as the initial set and then concatenate all users’ extracted ideas to the baseline set.

### Production equalization for AI-assisted creators

To decouple productivity effects from other underlying impacts of AI, we must consider a scenario where AI-assisted creators do not experience a productivity boost over their nonadopter counterparts. To accomplish this, we estimate the production-equalized convex hulls for the AI-assisted creators by assuming that they follow the same productivity trends as the nonadopters after AI is released. We take the artifacts that the AI-assisted creators produce in the post-Midjourney period and randomly sample the same number of artifacts as the amount produced by the matched nonadopters post-Midjourney. On average, this reduces the production quantity of the AI-assisted creators by approximately 48% of their original quantity. We use the same bootstrapped matching procedure as in the main estimation for 200 draws to also construct a comparable nonadopter convex hull such that we can recover the number of vertices that define each convex hull.

With 200 permutations of both AI-assisted creators’ and nonadopters’ hulls, we bootstrap from the observed instantiations of the convex hulls to obtain the empirical distribution of the number of vertices for both AI-assisted creators and nonadopters. Over each period, we compute the difference between the AI-assisted creators’ and nonadopters’ number of vertices that define the idea frontier to obtain the difference in vertex discovery rates between the two groups. Further, we perform this bootstrapping process for the observed data from the main results, the production-equalized AI-assisted creator data, and its matched nonadopter counterpart, with volume-equalized AI-assisted hive minds and masterminds (the bottom 80% and top 20% of vertex contributors, respectively; see Fig. 2), and lastly with volume-equalized low and high-exploration users before adopting AI classified on the basis of whether the average within-user distance empirical cumulative distribution function (ECDF) of all artifacts produced before AI is below or above the mean distance ECDF.

## Supplementary Materials

### This PDF file includes:

Supplementary Text Figs. S1 to S6 Tables S1 to S4 References
