# Supplementary material for: Who expands the human creative frontier with generative AI: Hive minds or masterminds?
Source: Sci Adv. 2025 Sep 3;11(36):eadu5800. doi: 10.1126/sciadv.adu5800 (PMC12407059; doi:10.1126/sciadv.adu5800)
Supplement: Supplementary file 1 — Supplementary Text Figs. S1 to S6 Tables S1 to S4 References [file sciadv.adu5800_sm.pdf]

Science Advances
AAAS

Supplementary Materials for

Who expands the human creative frontier with generative AI: Hive minds or masterminds?

Eric B. Zhou *et al.*

Corresponding author: Eric B. Zhou, ebzhou@bu.edu

Sci. Adv. **11**, eadu5800 (2025)
DOI: 10.1126/sciadv.adu5800

This PDF file includes:

Supplementary Text
Figs. S1 to S6
Tables S1 to S4
References

Supplementary Text

Identification Strategy

There are two notable threats to identifying a causal relationship between adoption of AI tools and idea space expansion. First, the initial idea space between the AI-assisted creators and their matched non-adopters should be comparable in the pretreatment period, suggesting that they have "similar" ideas prior to any treatment events. If, for example, AI-assisted creators have a smaller idea space during the pretreatment periods, then any post-Midjourney expansions of the idea space may be attributed to individuals seizing low-hanging fruit ideas that simply were not explored by chance up to that point. Thus, any such expansions may not reflect meaningful contributions. Second, the sheer volume difference in the AI-assisted creator and non-adopter populations (4,395 versus 26,681) means that simply by chance, there is likely to be more diverse creators among the non-adopters than the AI-assisted creators which is directly correlated with idea space size. This effect may also manifest among users who are exceptionally productive where the volume of artworks produced is also correlated with idea space expansion by chance. To elaborate, if we consider instances of creativity as individuals sampling from an idea distribution in the way of blind variation (*?*), then a greater volume of random draws either from the same distribution or more diverse idea distributions is more likely to produce an extreme value which represents a divergent idea that will expand the convex hull.

To help address these concerns and provide a causal interpretation for our analysis, we leverage propensity score matching to mitigate unobservable confounding. Specifically, we anticipate that users' behaviors on the platform are correlated with their propensity to self-select into adopting text-to-image tools. For example, a user who is already successful in producing organic artworks likely has less incentive to adopt AI tools, whereas a low-productivity, less successful artist may be more likely to adopt such tools. Further, we want to account for the possibility that a user with a broad-spanning network on the platform may be the beneficiary of idea diffusion. Using the non-parametric approach XGBoost (*47*), we model selection as a function of the number of followers and average per-period productivity prior to any AI tools being released in February 2022. We leverage the propensity scores to match AI-assisted creators to their nearest neighbor counterfactual non-adopter with a caliper distance of 0.1.

Then, we use a continuous difference-in-difference (DID) approach (*48*) allowing for time-varying treatment effects over an exogenous treatment event with matched cohorts, focusing on the periods corresponding to the major model releases. A causal interpretation of our estimates requires that we satisfy the parallel trends assumption in that the outcome variable would have followed similar trends in the AI-assisted creators and non-adopters in the absence of any model release. We examine the continuous expansion of the convex hull corresponding to artists who adopt generative AI tools at any point in time during the observation period compared to artists who never adopt such tools. We estimate the econometric specification in Equation S1 where y_{it} corresponds to the convex hull metric for matched cohort i in period t. We include fixed effects D where $D = 1$ corresponds to AI-assisted creators and $D = 0$ to the non-adopters to capture any potential systematic differences between the two groups as well as cohort fixed effects γ_i to control for potential idiosyncratic differences between matched cohorts, and monthly fixed effects τ_t to account for time-varying trends. The variables of interest are the interactions between treatment D and τ_t which capture relative differences between the AI-assisted creators' and non-adopters' idea spaces over time via β_t. We estimate a coefficient for each time period t to observe the evolution of the treatment effect. The outcome variables include idea space volume, surface area, and the number of unique users who own at least one vertex of the idea space. In terms of volume and area, positive significant interaction terms signify that the AI-assisted creators' idea frontier is significantly larger than that of the non-adopters' in the given month.

$$y_{it} = D + \tau_t + \gamma_i + \beta_t(D \cdot \tau_t) + \varepsilon_{it} \tag{S1}$$

We also note that we are measuring the impact of individual contributions on the aggregate idea frontier, not specifically the share that an artist contributes to the convex hull. Intuitively, this means that the estimates reflect artists expanding the idea frontier when considering all users' contributions at once - the existing "universe of ideas" - rather than individual artists filling out the space they uniquely operate in.

In order to achieve variation in the data such that we can recover standard errors and show the results are robust to sampling and matching, we perform a bootstrapping procedure where we first randomly sample 300 AI-assisted creators from the set of all possible treatment users. Next, we obtain the counterfactual non-adopter units via one-to-one nearest neighbor matching without

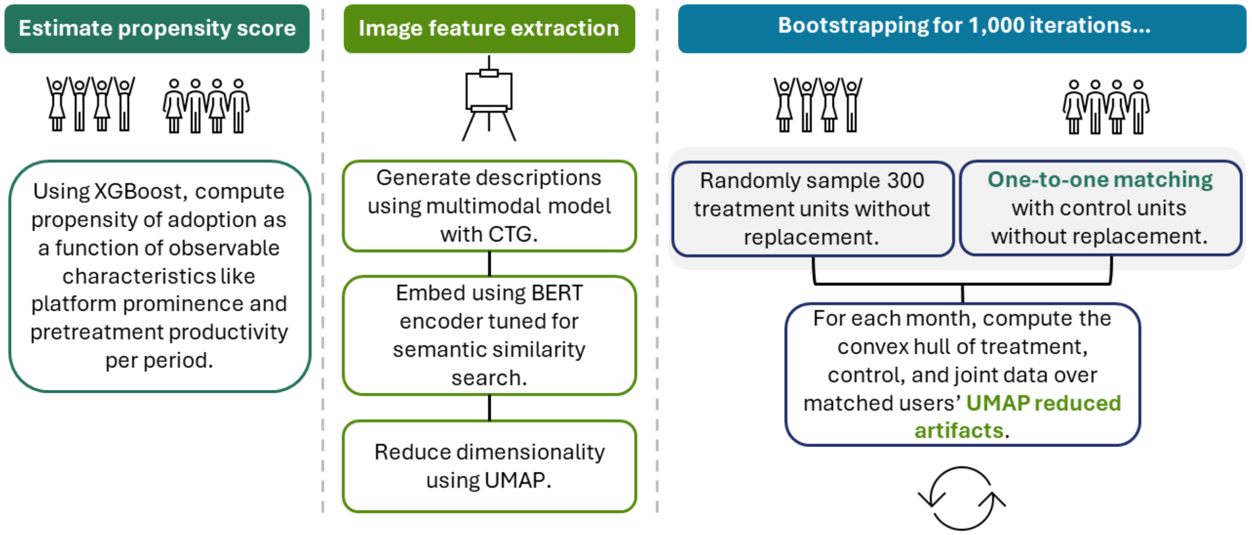

Fig. S1: Overview of empirical strategy

replacement. We use one-to-one matching without replacement for several reasons: (1) we can account for artist volume which may otherwise affect idea diversity, (2) this mitigates production volume effects via how we model the propensity score over the average productivity per period prior to any model release event, and (3) only unique contributions to the idea space can have an impact on the convex hull; otherwise, sampling with replacement may introduce volume disparities. We use a fixed cohort size without considering the entry of new creators to the platform in later periods because we are focally concerned with constructing comparable cohorts from users who are likely to exhibit the same production behaviors for the entire data period to satisfy the parallel trends assumption while minimizing potential bias from idea distribution diversity that may arise from a changing cohort composition, not necessarily with the impact of entrants on the idea frontier.

Then, we compute the convex hull for each month between August 2021 and December 2023 and obtain the volume, surface area, number of vertices, and number of unique users corresponding to the vertices of the convex hulls for AI-assisted creators, non-adopters, and the joint set of artifacts. We repeat this process to generate 1,000 permutations, repeatedly shuffling and sampling from the AI-assisted creators and re-matching to counterfactual non-adopter units to generate 1,000

unique matched cohorts. This sampling strategy allows us to produce as many unique cohorts of AI-assisted creators and non-adopters as the number of iterations, where cohorts can have overlap in individuals across cohorts. This way, we can robustly show our estimates are not a potential artifact of the sampling procedure while also inviting natural idea diversity into our samples. Additionally, we choose to sample from the same complete AI-assisted creator pool each iteration so that we can achieve better coverage of the data using the larger control group. An overview of the data preparation procedure is detailed in Fig. S1. Estimates can be found in Tables S3 and S4.

Because our unit of analysis is creator cohorts, we do not explicitly address individual-level selection into adoption. We structure the main analysis this way for several reasons. First, ideas contributed in the past by one creator should not be considered as a novel contribution by any user in later periods. By constructing an aggregate idea frontier, we account for this case while also directly addressing our intended research question: whether AI-assistance helps creators discover ideas that are unforeseen by others. Second, if we were to use an event study DID framework where we measure individual convex hull performance over time relative to time of adoption, then we are only measuring individual-level idea discovery while remaining agnostic to true H-creativity and the possibility that ideas external to an individual may have been discovered before. This would effectively capture P-creative activities at the individual-level, which often will not be H-creative contributions at the cohort-level yet the estimates can reflect high degrees of individual idea discovery. This individual-level analysis was also initially studied in (*10*) using an event study framework. Still, we do provide empirical evidence of how AI assistance offers individual-level performance gains in terms of the volume effect and human-AI effect that may lead to H-creativity observed in the main result. For this study, an appropriate interpretation of the estimates should be the Average Treatment Effect on the Treated (ATT) where the treatment unit is a cohort of AI-assisted creators rather than the Average Treatment Effect (ATE).

Note that using fixed cohorts in each bootstrapped sample means that even if a creator begins using AI and is thus labeled as a treated unit but later stops using it, that creator will continue to be considered a member of the AI-assisted creator cohort. While we show evidence that AI usage persists in the long-run after the initial adoption period (see the following section on Intensity of AI Usage), if a user does cease to use text-to-image tools, then on average they should arguably return to a creativity level around that of a non-adopter. Given that the convex hull is constructed

such that it only expands when a new contribution exists outside of the previous period's convex hull, these users who stop using AI should have less ability to expand the idea frontier, particularly if the effect identified in the main estimation holds true. Thus, such cases of adopters later halting AI usage should, on average, attenuate our estimates, making our results a conservative estimate of the true treatment effect. Then, the result should follow those found in our sensitivity analysis below.

We further caveat that we cannot feasibly capture the "entire universe" of ideas, suggested by the formal definition of H-creativity as originally posed by Boden (*20*), as that is neither computationally feasible nor do we have the data to model the entire idea space. Still, our study provides a second-best approach that poses the following causal framing: given two comparable matched cohorts of AI-assisted creators and non-adopters, does the adoption of generative AI tools accelerate idea frontier expansion, which is suggestive of at least P-creative contributions and potentially H-creative ideas? Within the matched cohorts, any such contribution that leads to idea frontier expansion is necessarily P-creative and a candidate H-creative idea. For the sake of simplicity, we refer to any frontier-expanding contribution as H-creative within the respective cohort. Lastly, this interpretation is agnostic to the usefulness or "value" of a creative contribution and instead focuses solely on the novelty of an idea. The notion of "value" is highly contextual, and given we do not observe many contextual factors in our empirical setting, we leave the thorough investigation of this feature up to future research.

Intensity of AI Usage Over Time

One potential concern emerges when considering if individual-level experimentation with AI leads to prolonged AI usage or if creators simply stop using the tool in the long-run. If the identified AI-assisted creators in our sample abandon the tool after the early adoption periods, then we may not conclude that text-to-image tools have a lasting impact on humans' creative process leading to idea discovery. We provide descriptive evidence in Fig. S2 that individuals within the AI-assisted creator population continue to publish large quantities of AI-labeled artworks each month on average. To identify AI-assisted images, we utilize artwork-level title and tags to match on relevant keywords like "AI art, Midjourney, DALL-E, Stable Diffusion." In aggregate, this amounts to approximately 20 AI-assisted artworks posted per month per user. This suggests that AI-assisted creators are not

simply dropping AI usage after early experimentation, but instead remain highly productive in contributing AI-assisted artifacts.

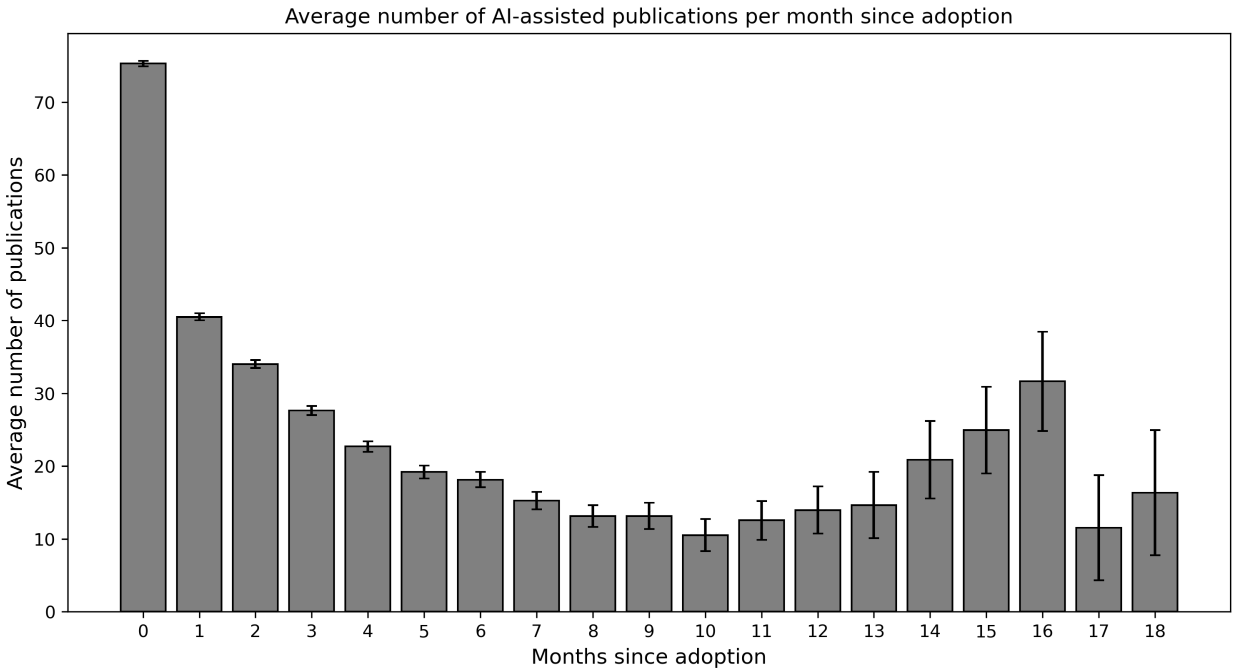

Fig. S2: **Average number of AI-assisted artworks published per month relative to adoption across the AI-assisted creator population.**

Sensitivity Analysis: Control Group Contamination

We consider the possibility that some users who are actually adopters choose not to disclose their treatment status, thus contaminating the control group. To assess the robustness of our treatment effect estimates in the presence of misidentification of adopters, leading to control group contamination, we run a sensitivity analysis that allows for varying degrees of unidentified treatment spillover into the control group. Specifically, we systematically introduce contamination of known AI-assisted creators by allowing up to 40% of the control group to be contaminated by randomly sampled treatment units.

We test contamination ratios starting at 10% and incrementing by 5% up to a maximum ratio of 40%. For each contamination ratio, we follow the main bootstrapped matching estimation procedure for 200 iterations while randomly sampling among AI-assisted creators not included in

that iteration's treatment cohort to serve as contamination in the control group. We then re-estimate the event study DID. Our findings, shown in Fig. S3 indicate that the estimated treatment effects remain robust up to 30% contamination where the original effect size is attenuated by up to 50% in the long-run. Beyond this contamination ratio, the treatment effect is no longer significant. Thus, assuming that the originally identified treatment effect is valid, we see that unidentified AI-assisted creators contaminating the control group weakens the treatment effect. This is due to the contaminated users exhibiting treatment-like behavior where, on average, these users are more likely to contribute novel ideas.

Intuitively, if we assume that our identified effect where AI-assisted creators begin accelerating idea discovery in the long-run holds true on average, then if unidentified treatment users exist in the control cohort, we should expect that our main results will be attenuated. Thus, any such control group contamination should lead to underestimation, and our results can be considered a conservative estimate of the true treatment effect.

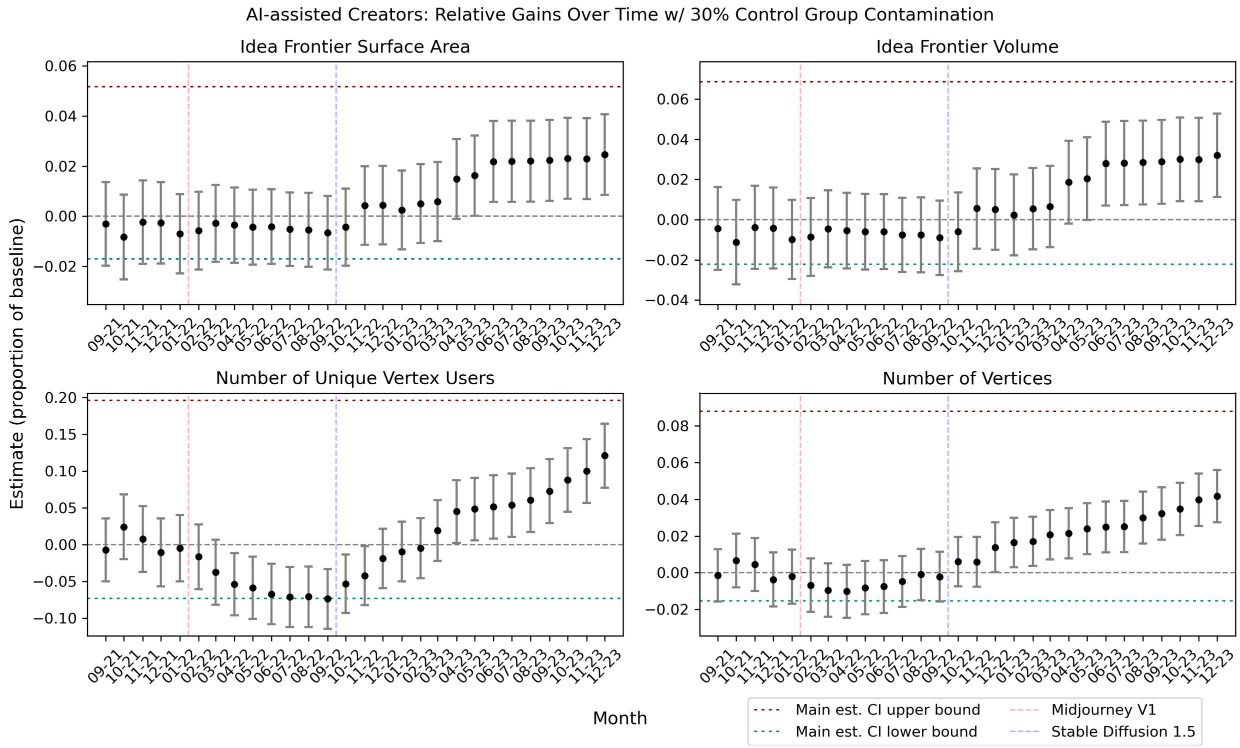

Fig. S3: **Event study difference-in-differences model measuring the impact of text-to-image model releases on AI-assisted creators' idea frontier surface area, volume, number of vertices, and number of unique users who own a vertex relative to the non-adopters with up to 30% control group contamination.** The red and blue dashed lines denote the maximum and minimum confidence interval bounds from the main estimation results. The control contaminated estimates are attenuated compared to the main results.

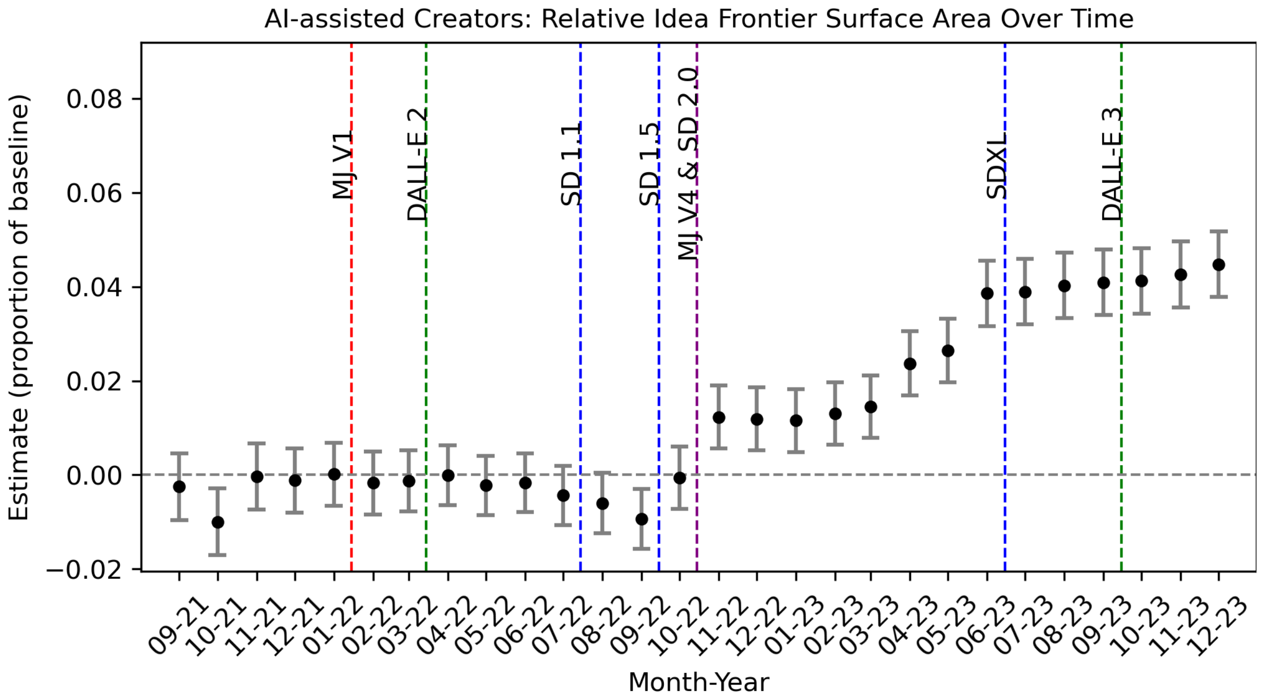

Fig. S4: **Same as Fig. 1A, but for surface area.**

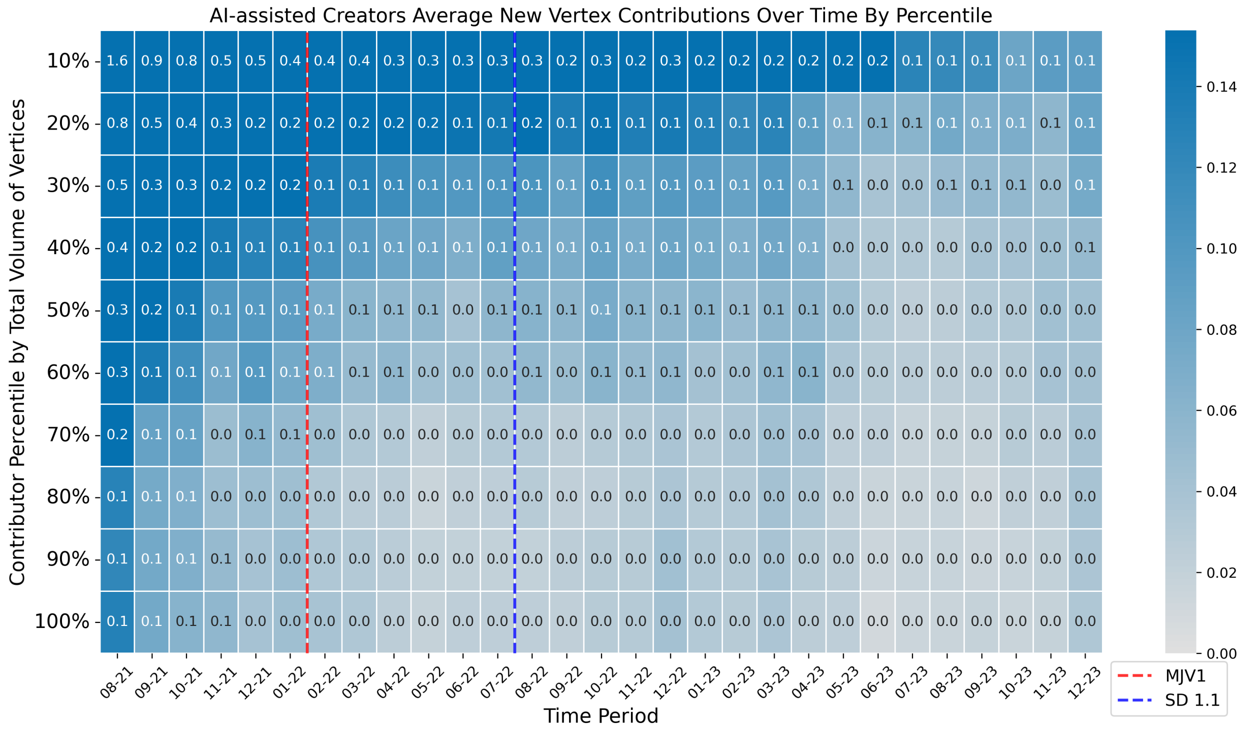

Fig. S5: **Heatmap for the difference in the number of vertex contributions for AI-assisted creators grouped by percentiles of total vertices contributed**.

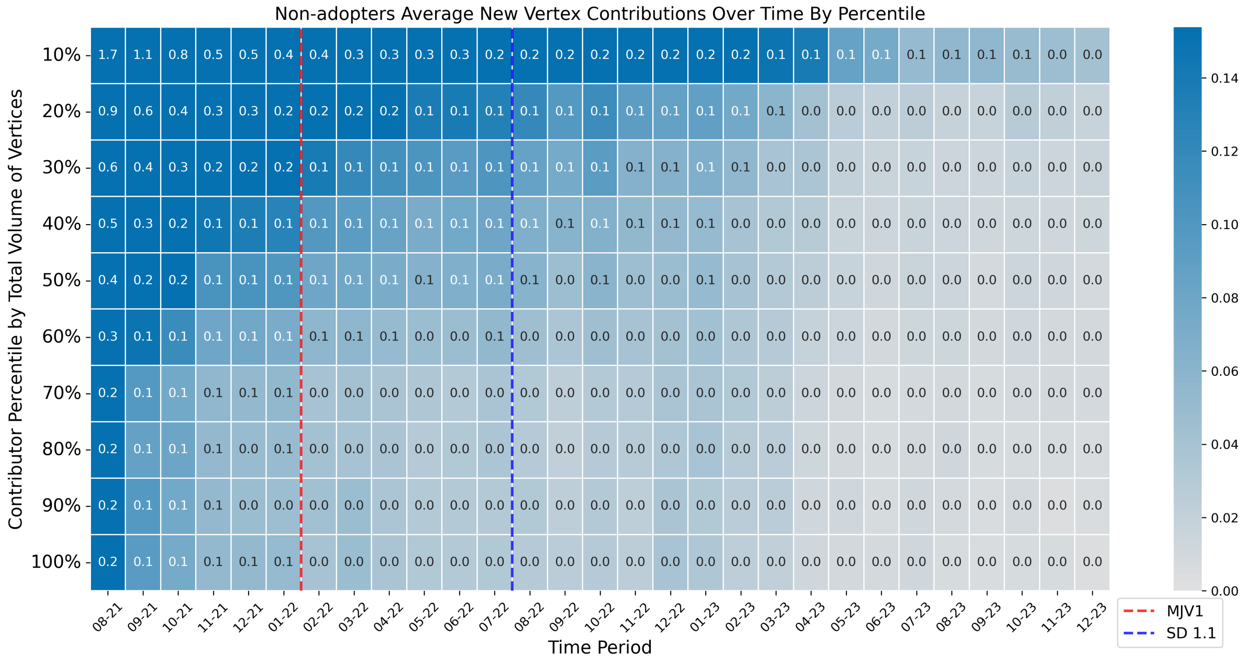

Fig. S6: **Same as Fig. S5, but for non-adopters.**

Model	Version	Release Month
Midjourney	V1	February 2022
	V4	November 2022
Stable Diffusion	1.1	August 2022
	1.5	October 2022
	2.0	November 2022
	XL	July 2023
DALL-E	2	April 2022
	3	October 2023

Table S1: Summary of major model release months

	N	Mean	SD	Min	Max
AI-assisted creators	4,395				
non-adopters	26,681				
Total number of artworks	31,076	387.74	665.19	1	18,990
Average posts per month	31,076	7.30	16.15	1	1,061
Profile page views	31,076	35,132.91	78,529.13	49	1,787,366
Profile comments	31,076	381.17	1,219.81	0	34,299
Followers	31,076	633.28	1,276.030	0	23,665

Table S2: Descriptive Statistics

Table S3: Event study difference-in-differences model measuring the effect of text-to-image model releases on idea frontier surface area and volume for AI-assisted creators over time.

	Surface Area	Volume
	(1)	(2)
Intercept	22356.335***	19384.464***
	(108.573)	(123.323)
Treatment	-443.873***	-450.916***
	(54.197)	(57.719)
Sep '21	-54.242	-74.576
	(79.229)	(84.144)
Jan '22	3.617	-10.105
	(75.163)	(80.546)
Feb '22 (MJ V1)	-37.748	-58.827
	(74.365)	(79.916)
Jun '22	-36.386	-32.592
	(69.848)	(75.604)
Oct '22 (SD 1.5)	-13.267	0.352
	(73.739)	(80.993)
Nov '22	269.436***	315.944***
	(75.095)	(82.753)
Dec '22	260.772***	294.278***
	(74.936)	(82.683)
Jan '23	253.616***	289.221***
	(74.749)	(82.538)
Jun '23	845.385***	966.836***
	(77.859)	(86.658)
Dec '23	981.211***	1129.693***
	(77.850)	(86.723)
Cohort Fixed Effects	Yes	Yes
Month Fixed Effects	Yes	Yes
Cohort-Month Observations	28,971	28,971
R^2	0.749	0.752

Note: *p<0.05; **p<0.01; ***p<0.001
All period variables denote the interaction between period and treatment.
Heteroscedastic robust standard errors are reported.
Only estimates for start and end dates as well as noteworthy quarterly periods are shown.

Table S4: Event study difference-in-differences model measuring the effect of text-to-image model releases on idea frontier number of unique vertex users for AI-assisted creators over time.

	Number of Vertices	Unique Vertex Users
	(1)	(2)
Intercept	296.473***	44.671***
	(1.533)	(0.702)
Treatment	0.927	-3.397***
	(0.591)	(0.250)
Sep ’21	-1.711	-0.399
	(0.889)	(0.365)
Jan ’22	-1.810	1.832***
	(0.951)	(0.379)
Feb ’22 (MJ V1)	-2.697**	1.444***
	(0.941)	(0.379)
Jun ’22	-1.067	-1.184***
	(0.883)	(0.357)
Oct ’22 (SD 1.5)	3.565***	-1.628***
	(0.862)	(0.354)
Nov ’22	3.569***	-1.382***
	(0.860)	(0.352)
Dec ’22	6.751***	-0.572
	(0.864)	(0.353)
Jan ’23	7.247***	-0.876*
	(0.871)	(0.356)
Jun ’23	13.631***	2.813***
	(0.872)	(0.362)
Dec ’23	24.374***	7.363***
	(0.894)	(0.375)
Cohort Fixed Effects	Yes	Yes
Month Fixed Effects	Yes	Yes
Cohort-Month Observations	28,971	28,971
R^2	0.827	0.855

Note: *p<0.05; **p<0.01; ***p<0.001
All period variables denote the interaction between period and treatment.
Heteroscedastic robust standard errors are reported.
Only estimates for start and end dates as well as noteworthy quarterly periods are shown.

REFERENCES AND NOTES

1. S. Peng, E. Kalliamvakou, P. Cihon, M. Demirer, The impact of AI on developer productivity: Evidence from GitHub Copilot. arXiv:2302.06590 [cs.SE] (2023).

2. F. Dell'Acqua, E. Mc Fowland III, E. Mollick, H. Lifshitz-Assaf, K. C. Kellogg, S. Rajendran, L. Krayer, F. Candelon, K. R. Lakhani, "Navigating the jagged technological frontier: Field experimental evidence of the effects of AI on knowledge worker productivity and quality," working paper no. 24-013, Harvard Business School, Boston, MA, September 2023.

3. S. Noy, W. Zhang, Experimental evidence on the productivity effects of generative artificial intelligence. *Science* **381**, 187–192 (2023).

4. S. Huang, P. Grady, GPT-3, Generative AI: A Creative New World, https://sequoiacap.com/article/generative-ai-a-creative-new-world/ (2022).

5. R. Rombach, A. Blattmann, D. Lorenz, P. Esser, B. Ommer, High-resolution image synthesis with latent diffusion models, in *2022 IEEE/CVF Conference on Computer Vision and Pattern Recognition (CVPR)* (IEEE, 2022), pp. 10674–10685.

6. L. Zhang, A. Rao, M. Agrawala, Adding conditional control to text-to-image diffusion models. arXiv:2302.05543 [cs.CV] (2023).

7. C. Meng, Y. He, Y. Song, J. Song, J. Wu, J.-Y. Zhu, S. Ermon, SDEdit: Guided image synthesis and editing with stochastic differential equations. arXiv:2108.01073 [cs.CV] (2022).

8. R. Ranftl, K. Lasinger, D. Hafner, K. Schindler, V. Koltun, Towards robust monocular depth estimation: Mixing datasets for zero-shot cross-dataset transfer. arXiv:1907.01341 [cs.CV] (2020).

9. A. Hertzmann, Can computers create art? *Arts* **7**, 18 (2018).

10. E. Zhou, D. Lee, Generative artificial intelligence, human creativity, and art. *PNAS Nexus* **3**, pgae052 (2024).

11. L. Meincke, K. Girotra, G. Nave, C. Terwiesch, K. T. Ulrich, Using large language models for idea generation in innovation. Social Science Research Network 4526071 [Preprint] (2024); https://doi.org/10.2139/ssrn.4526071.

12. J. Joosten, V. Bilgram, A. Hahn, D. Totzek, Comparing the ideation quality of humans with generative artificial antelligence. *EEE Eng. Manag. Rev.* **52**, 153–164 (2024).

13. J. Haase, P. H. P. Hanel, Artificial muses: Generative artificial intelligence chatbots have risen to human-level creativity. *J. Creat.* **33**, 100066 (2023).

14. L. Meincke, E. R. Mollick, C. Terwiesch, Prompting diverse ideas: Increasing AI idea variance (2024).

15. A. R. Doshi, O. P. Hauser, Generative AI enhances individual creativity but reduces the collective diversity of novel content. *Sci. Adv.* **10**, eadn5290 (2024).

16. M. Koivisto, S. Grassini, Best humans still outperform artificial intelligence in a creative divergent thinking task. *Sci. Rep.* **13**, 13601 (2023).

17. T. Chakrabarty, P. Laban, D. Agarwal, S. Muresan, C.-S. Wu, Art or artifice? Large language models and the false promise of creativity. arXiv:2309.14556 [cs.CL] (2024).

18. C. Stevenson, I. Smal, M. Baas, R. Grasman, H. van der Maas, Putting GPT-3's creativity to the (alternative uses) test. arXiv:2206.08932 [cs.AI] (2022).

19. L. Boussioux, J. N. Lane, M. Zhang, V. Jacimovic, K. R. Lakhani, The crowdless future? Generative AI and creative problem-solving. *Org. Sci.* **35**, 1589–1607 (2024).

20. M. A. Boden, Creativity and artificial intelligence. *Artif. Intell.* **103**, 347–356 (1998).

21. M. A. Boden, Computer models of creativity. *AI Mag.* **30**, 23–34 (2009).

22. M. A. Boden, *The Creative Mind: Myths and Mechanisms* (Routledge, 2004).

23. E. J. Hu, Y. Shen, P. Wallis, Z. Allen-Zhu, Y. Li, S. Wang, L. Wang, W. Chen, LoRA: Low-rank adaptation of large language models. arXiv:2106.09685 [cs.CL] (2021).

24. E. Brynjolfsson, D. Rock, C. Syverson, The productivity J-curve: How intangibles complement ceneral purpose technologies. *Am. Econ. J. Macroecon.* **13**, 333–372 (2021).

25. D. W. Galenson, Quantifying Artistic Success: Ranking French Painters—and Paintings—from Impressionism to Cubism. *Hist. Methods.* **35**, 5–19 (2002).

26. K. Bender, Distant Viewing in Art History. A case study of artistic productivity. *International Journal for Digital Art History* **1**, 100–110 (2015).

27. S. P. Fraiberger, R. Sinatra, M. Resch, C. Riedl, A.-L. Barabási, Quantifying reputation and success in art. *Science* **362**, 825–829 (2018).

28. A. Abadie, A. Diamond, J. Hainmueller, Synthetic Control methods for comparative case studies: Estimating the effect of California's tobacco control program. *J. Am. Stat. Assoc.* **490**, 493–505 (2010).

29. E. Ben-Michael, A. Feller, J. Rothstein, The augmented synthetic control method. *J. Am. Stat. Assoc.* **116**, 1789–1803 (2021).

30. T. Eloundou, S. Manning, P. Mishkin, D. Rock, GPTs are GPTs: Labor market impact potential of LLMs. *Science* **384**, 1306–1308 (2024).

31. E. Hermann, S. Puntoni, Artificial intelligence and consumer behavior: From predictive to generative AI. *J. Bus. Res.* **180**, 114720 (2024).

32. M. Shin, J. Kim, B. van Opheusden, T. L. Griffiths, Superhuman artificial intelligence can improve human decision-making by increasing novelty. *Proc. Natl. Acad. Sci. U.S.A.* **120**, e2214840120 (2023).

33. Z. Epstein, A. Hertzman, the Investigators of Human Creativity, Art and the science of generative AI. *Science* **380**, 1110–1111 (2023).

34. D. T. Campbell, Blind variation and selective retention in creative thought as in other knowledge processes. *Psychol. Rev.* **67**, 380–400 (1960).

35. D. K. Simonton, Creativity as blind variation and selective retention: Is the creative process Darwinian? *Psychol. Inq.* **10**, 309–328 (1999).

36. W. Orwig, L. Bellaiche, S. Spooner, A. Vo, Z. Baig, A. Ragnhildstveit, P. Seli, Using AI to generate visual art: Do individual differences in creativity predict AI-assisted art quality? *Creat. Res. J.*, 1–12 (2024).

37. L. Brinkmann, F. Baumann, J.-F. Bonnefon, M. Derex, T. F. Müller, A.-M. Nussberger, A. Czaplicka, A. Acerbi, T. L. Griffiths, J. Henrich, J. Z. Leibo, R. McElreath, P.-Y. Oudeyer, J. Stray, I. Rahwan, Machine culture. *Nat. Hum. Behav.* **7**, 1855–1868 (2023).

38. S. Schockaert, H. Prade, Interpolative and extrapolative reasoning in propositional theories using qualitative knowledge about conceptual spaces. *Artif Intell* **202**, 86–131 (2013).

39. D. Yeverechyahu, R. Mayya, G. Oestreicher-Singer, The impact of large language models on open-source innovation: Evidence from GitHub copilot. arXiv:2409.08379 [cs.SE] (2024).

40. N. Goodman, *Languages of Art* (Hackett Publishing, 1976).

41. R. Wollheim, Nelson Goodman's languages of art. *J. Philos.* **67**, 531–539 (1970).

42. J. Li, D. Li, S. Savarese, S. Hoi, BLIP-2: Bootstrapping language-image pretraining with frozen image encoders and large language models. arXiv:2301.12597 [cs.CV] (2023).

43. N. Reimers, I. Gurevych, Sentence-BERT: Sentence embeddings using Siamese BERT-networks. arXiv:1908.10084 [cs.CL] (2019).

44. L. McInnes, J. Healy, J. Melville, UMAP: Uniform Manifold Approximation and Projection for dimension reduction. arXiv:1802.03426 [stat.ML] (2020).

45. M. Grootendorst, BERTopic: Neural topic modeling with a class-based TF-IDF procedure. arXiv:2203.05794 [cs.CL] (2022).

46. N. Garcia, G. Vogiatzis, How to read paintings: Semantic art understanding with multi-modal retrieval, in *Computer Vision – ECCV 2018 Workshops*, L. Leal-Taixé, S. Roth, Eds. (Springer International Publishing, 2019) vol. 11130, pp. 676–691.

47. T. Chen, C. Guestrin, XGBoost: A scalable tree boosting system, in *Proceedings of the 22nd ACM SIGKDD International Conference on Knowledge Discovery and Data Mining* (2016), pp. 785–794.

48. J. D. Angrist, J.-S. Pischke, *Mostly Harmless Econometrics: An Empiricist's Companion* (Princeton Univ. Press, 2009).
